# EGFR-Mutated Non-Small Cell Lung Cancer and Resistance to Immunotherapy: Role of the Tumor Microenvironment

**DOI:** 10.3390/ijms23126489

**Published:** 2022-06-10

**Authors:** Clelia Madeddu, Clelia Donisi, Nicole Liscia, Eleonora Lai, Mario Scartozzi, Antonio Macciò

**Affiliations:** 1 Department of Medical Sciences and Public Health, Medical Oncology Unit, “Azienda Ospedaliero Universitaria” of Cagliari, University of Cagliari, 09100 Cagliari, Italy; clelia.madeddu@unica.it (C.M.); nikina310788@gmail.com (N.L.); ele.lai87@gmail.com (E.L.); marioscartozzi@gmail.com (M.S.); 2 Gynecologic Oncology Unit, ARNAS G. Brotzu, Department of Surgical Sciences, University of Cagliari, 09100 Cagliari, Italy; clelia.madeddu@tiscali.it

**Keywords:** EGFR mutations, non-small cell lung cancer, immunotherapy resistance, tumor microenvironment, tumor-associated macrophages, tyrosine kinase inhibitor

## Abstract

Lung cancer is a leading cause of cancer-related deaths worldwide. About 10–30% of patients with non-small cell lung cancer (NSCLC) harbor mutations of the EGFR gene. The Tumor Microenvironment (TME) of patients with NSCLC harboring EGFR mutations displays peculiar characteristics and may modulate the antitumor immune response. EGFR activation increases PD-L1 expression in tumor cells, inducing T cell apoptosis and immune escape. EGFR-Tyrosine Kinase Inhibitors (TKIs) strengthen MHC class I and II antigen presentation in response to IFN-γ, boost CD8+ T-cells levels and DCs, eliminate FOXP3+ Tregs, inhibit macrophage polarization into the M2 phenotype, and decrease PD-L1 expression in cancer cells. Thus, targeted therapy blocks specific signaling pathways, whereas immunotherapy stimulates the immune system to attack tumor cells evading immune surveillance. A combination of TKIs and immunotherapy may have suboptimal synergistic effects. However, data are controversial because activated EGFR signaling allows NSCLC cells to use multiple strategies to create an immunosuppressive TME, including recruitment of Tumor-Associated Macrophages and Tregs and the production of inhibitory cytokines and metabolites. Therefore, these mechanisms should be characterized and targeted by a combined pharmacological approach that also concerns disease stage, cancer-related inflammation with related systemic symptoms, and the general status of the patients to overcome the single-drug resistance development.

## 1. Introduction

Lung cancer is a leading cause of cancer-related deaths worldwide [1]. About 10–30% of patients with non-small cell lung cancer (NSCLC) harbor mutations, mainly in exons 18–21, of the EGFR gene, which is considered an oncogenic driver [2]. Among these, exon 19 deletion mutation (p. E746–A750del) and exon 21-point mutation (p.L858R) are the most frequent, accounting for more than 85% of EGFR mutations. Other EGFR mutations are considered uncommon [3].

EGFR tyrosine kinase inhibitors (TKIs) represent the first-line treatment and targeted therapy for patients with metastatic NSCLC harboring EGFR mutations [3]. However, drug resistance occurs in these patients, and due to the failure of the immune surveillance, cancer cell growth continues [4,5].

Immunotherapy with programmed cell death protein 1 (PD-1) and programmed death-ligand 1 (PD-L1) inhibitors is considered a promising treatment strategy for NSCLC. However, current evidence suggests that such immunotherapy is not beneficial for patients with NSCLC carrying EGFR mutations. Many preclinical and clinical studies, as well as a meta-analysis of five clinical trials (Checkmate 017 and 057, Keynote 010, OAK, and POPLAR)*,* showed poor effectiveness of PD-1 inhibitors in patients with EGFR-mutated NSCLC [6,7]. Nonetheless, according to some studies, immunotherapy is effective in patients with uncommon EGFR mutations.

In this review, we will discuss the importance of the tumor microenvironment (TME) and immune response in lung cancer evolution and prognosis, the role of EGFR mutations in influencing the immune response and resistance to immunotherapy, dynamic changes in the TME and immune cells during TKI treatment, strategies for overcoming resistance to immunotherapy, and the rationale for combining TKI treatment and immunotherapy in EGFR-mutated NSCLC.

## 2. Role of TME in Lung Cancer Evolution and Prognosis

**Figure 1:**
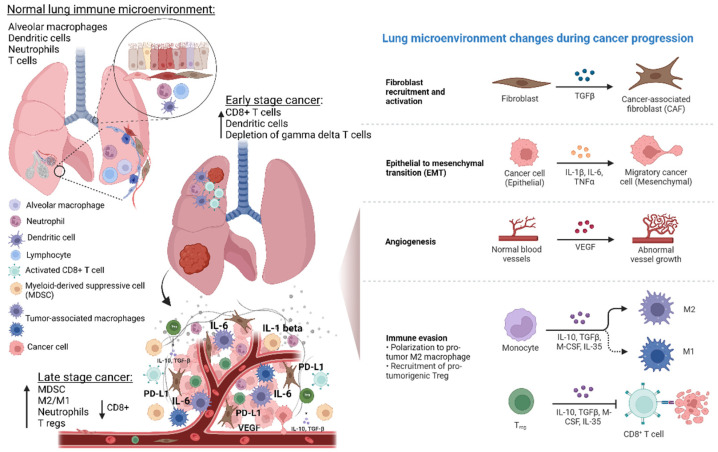
Lung microenvironment changes from physiological state to NSCLC development and progression. Normal lung microenvironment includes epithelial cells, smooth muscle cells, fibroblast, endothelial cells, and immune cells such as dendritic cells, neutrophils, T cells, and alveolar macrophages. The latter contribute to maintain immunological homeostasis, but they can also promote inflammation, and thus, development of premalignant lung lesions and carcinogenesis. During NSCLC development, the tumor microenvironment (TME) changes, thus contributing to inflammation angiogenesis, immune modulation, and therefore promoting NSCLC progression, metastasis, and prognosis. In particular, the immune TME, through specific reprogramming and modulation of T cells, tumor-associated macrophages (TAMs) and myeloid cell populations exert crucial tumor-promoting or tumor-suppressing activities. The switch from tumor immune surveillance to cancer immune escape is characterized by the recruitment of regulatory T-cells (Tregs) and upregulation of MDSCs. In addition, TAMs (M1 and M2 polarized cells), and neutrophils play a key role in the mechanisms of immune escape. In particular, they contribute to create a proinflammatory TME that strongly affects the immune response efficiency. Abbreviations: MDSCs—myeloid-derived suppressor cells; IL—Interleukin; VEGF—vascular endothelial growth factor; PD-L—programmed death-ligand; TGF—tumor growth factor; CSF—colony-stimulating factor; M-CSF—macrophage-colony stimulating growth factor. Created with BioRender.com (https://biorender.com/, accessed on 17 May 2022).

The TME is composed of cancer cells, immune cells such as T cells, B cells, dendritic cells (DCs), myeloid-derived suppressor cells (MDSCs), tumor-associated macrophages (TAMs), carcinoma-associated fibroblasts, tumor vasculature, lymphatic tissue, as well as adipocytes, cytokines, and exosomes [8,9]. TME plays a crucial role in cancer development, progression, and metastasis owing to the intricate crosstalk between its components (Figure 1). T lymphocytes appear to mediate the switch from tumor immune surveillance to cancer immune escape through the recruitment of regulatory T-cells (Tregs) and upregulation of MDSCs [9]. In addition, it has been reported that macrophages and neutrophils are crucially involved in the mechanisms of immune escape and development of lung cancer. In particular, these cells create a proinflammatory background that strongly affects both carcinogenesis and immune-response efficiency [2].

In the normal physiological state, innate and adaptive immune cells catch and eradicate cancer cells through immunosurveillance [10]. However, cancer cells acquire the ability to escape from the antitumor immune response, and the dysregulated relationship between antagonistic effectors (i.e., CD8^+^ cytotoxic T-cells) and regulatory immune cells (i.e., Tregs) leads to a TME that can promote cancer development [11]. As part of the TME, TAMs play an active role in tumorigenesis. Macrophages constitute the majority of the cells in the immune infiltrate in tumors, and they have extremely varying effects on tumorigenesis, depending on their phenotype within the TME [12]. As key innate immune cells, they are involved in the immunological response against foreign cells and in tissue repair following an injury [13]. There are two types of activated macrophages: pro-inflammatory (M1) and anti-inflammatory (M2). M1 to M2 macrophage polarization is a plasticity phenomenon that occurs in various physiological situations, from bacterial infection to wound healing. When a wound fails to heal, it can become a chronic wound with persistent inflammation, which could create the basis for tumorigenesis [12]. It has been suggested that M1 macrophages eradicate cancer cells through reactive oxygen and nitrogen intermediates, and several proinflammatory cytokines, whereas M2 macrophages promote tumorigenesis and metastasis by secreting matrix-degrading enzymes, angiogenic factors, and immunosuppressive cytokines/chemokines, as well as by downregulating major histocompatibility complex class II (MHC II) and co-stimulatory ligands CD80 and CD86 [14,15,16]. Thus, M2 macrophages generally support tumor progression and metastasis. Interleukin (IL)-4, IL-10, and IL-13 secreted in response to the activation of signal transducer and transcriptional activator-3 (STAT3) and nuclear factor kappa B pathways contribute to M2 polarization. In addition, TAMs may cause immune suppression through immune checkpoint (PD-L1) induction and enhanced activation of specific metabolic pathways [13].

Consistently, TAM polarization in the TME has been reported to be associated with different prognoses of cancer patients, with the prevalence of M1 cells in the TME correlating with better progression-free and overall survival [17,18]. In contrast, the prevalence of M2 macrophages appears to be related to poor outcomes in lung cancer [19]. However, M1 macrophages, which produce reactive oxygen and nitrogen species, contribute to DNA damage, and may stimulate malignant transformation [13]. In lung cancer, M1 macrophages are found in cancer cell islets, and their presence is related to a good prognosis. In contrast, M2 macrophages are located in the tumor stroma and are associated with a poor prognosis. In the early stages of tumorigenesis, M1 macrophages consistently infiltrate tumor islets to prevent cancer progression. However, during cancer progression, M1 macrophages may acquire the M2 phenotype and start supporting tumor progression [15]. Additionally, TAM polarization leading to the prevalence of M2 macrophages in the TME can cause drug resistance [16]. Various mechanisms for the TAM-related drug resistance have been proposed. First, TAMs can promote the epithelial-to-mesenchymal transition by secreting transforming growth factor-β and tumor necrosis factor (TNF)-α and induce remodeling of the extracellular matrix through proteases such as matrix metalloproteinases (MMPs) [16,20,21].

Several studies have shown that TAM-secreted proteins such as MMPs, plasmin, urokinase-type plasminogen activator, vascular endothelial growth factor, IL-8, basic fibroblast growth factor, phosphatidylinositol-glycan biosynthesis class F protein, and gastrin-releasing peptide lead to therapeutic resistance and boost tumor angiogenesis [16,20]. Moreover, TAM-secreted immunosuppressive factors such as prostaglandin E2, IL-10, transforming growth factor-β, indoleamine-pyrrole 2,3-dioxygenase, chemokine C-C motif ligand (CCL) 17, CCL18, and CCL22 inhibit the Th1 immune response and also cause drug resistance [22,23,24].

### 2.1. Subsection

#### 2.1.1. Role of TAMs and Related Inflammation on the Efficacy of Immune Response and Immunotherapy

Tumor-associated macrophages (TAMs) are essential elements during the initial phase of the immune response due to their phagocytic capacity, ability to synthesize interferon (IFN), and interactions with helper and cytotoxic T lymphocytes. However, their persistent activation with consequent chronicization of inflammation, oxidative stress, and changes in metabolic pathways lead to the impairment of effective T-cell responses by causing T-cell exhaustion, a condition in which lymphocytes, even when activated, are non-functional and subsequently undergo programmed cell death [25]. In this regard, Mascaux et al. [26] demonstrated that in lung squamous cell carcinoma, the adaptive immune response was the strongest at the earliest cancer stages, whereas at the most advanced invasive stages, they observed increased expression levels of co-inhibitory molecules and suppressive cytokines, such as PD-L1, IL-10, and IL-6.

To understand the role of TAMs in the efficacy of immune response and immunotherapy, it is necessary to clarify the contribution of Tregs to cancer immunosuppression. Tregs are a specialized T-cell lineage that express the transcription factor FOXP3, which is crucial for Treg stabilization and stimulation of the Treg-specific gene expression profile necessary to prevent autoimmune reactions in normal conditions [27,28]. However, Tregs can switch their fate and phenotype under certain circumstances, such as inflammatory perturbations of the microenvironment. This is possible owing to changes in their gene expression program, which is characterized by the loss of FOXP3 expression and production of pro-inflammatory cytokines, and IFN-γ, which convert these cells into effector T-cells (Treg reprogramming). In a recent study by Di Pilato et al., an increase in IFN-γ levels favored the expression of PD-1 on effector T-cells and synthesis of PD-L1 by cancer cells and macrophages [29], thereby turning off CD4^+^CD25^−^ conventional effector T-cells, reactivating an immune escape mechanism and increasing macrophage activation [30]. Recently, Gallimore et al. highlighted an important therapeutic role of the induction of selective recruitment and modulation of different Tregs with different molecular profiles and functions in the TME [31]. Based on these observations, other authors observed that immunotherapy efficacy could be diminished by the inflammatory response caused by the reprogramming of Tregs [25]. Indeed, as already described above, various suppressive and counter-regulatory mechanisms may be involved in the lack of immunotherapy effectiveness, especially in the advanced stages of neoplastic disease. In patients with advanced cancer, specific changes in oxidative and glycolytic metabolic pathways during a chronic inflammatory response interfere with conventional T-cell activation and function and may be one of the reasons for the ineffectiveness of immunotherapy. Consequently, a combined strategy of modulating the activity of Tregs, pharmacological inhibition of chronic inflammation mediated by macrophages, and, at the same time, suppression of oxidative stress and positive regulation of metabolic imbalances could improve the effectiveness of modern immunotherapy. Reprogramming of Tregs has a dual effect: firstly, it immediately activates immunosurveillance, and secondly, it causes delayed, macrophage-mediated inflammation deleterious for the antineoplastic efficiency of the immune system response [32]. Furthermore, the loss of Treg activity shown in various in vivo and in vitro experimental models involving reprogramming of Tregs into IFN-γ-producing cells [33,34,35], is accompanied by M1 pro-inflammatory polarization of peritoneal macrophages with associated production of pro-inflammatory and immunosuppressive cytokines [36]. The persistent activation of macrophages does not favor sustained antitumor T-cell responses [37,38]. Following M1 macrophage polarization, other processes such as increased synthesis proinflammatory cytokines, production of reactive oxygen species, and changes in glucose and iron metabolism occur [39]. In particular, an iron-sequestering phenotype develops, which is characterized by intracellular iron accumulation and low iron release and availability (functional iron deficiency) [40]. Dysregulation of the iron metabolism impairs several vital cell processes, such as DNA and protein synthesis, enzyme activity, integrity of oxidative pathways, and cell proliferation, thereby resulting in a progressive loss of T-cell function when cancer advances [41].

Based on this evidence, a strategy that blocks chronic inflammation and Treg reprogramming should be considered in some patients depending on the cancer stage. Moreover, TAMs, specifically M1, are the main producers of IL-6, which plays a key role in modulating both tumor progression and immune escape through multiple mechanisms [25,42]. In particular, IL-6 is involved in lung cancer tumorigenesis, and its increased circulating levels have been associated with poor survival of patients with lung cancer [43]. In addition, IL-6 acts directly on lung epithelial cells via the nuclear factor kappa B signaling pathway when conditioned by exposure to carcinogens. Tobacco smoking is known to induce *KRAS* mutations and thereby stimulate IL-6 expression in the lung epithelium [44], promoting lung cancer cell proliferation and migration through the STAT3 pathway activation [45]. Exhausted tumor-associated CD8^+^ T lymphocytes are another source of IL-6 in lung cancer [46]. Moreover, IL-6 is one of the key cytokines driving the immunopathology caused by the prolonged non-specific inflammation contributing to the so-called “cytokine storm”, with related systemic symptoms and impairment of immune response. Indeed, IL-6 influences the effectiveness of the immune system in multiple ways. IL-6 can act as an activator or inhibitor of T-cell responses, depending on the duration of its activity; moreover, by inducing systemically specific derangements of energy metabolism, nutritional status, and symptoms as anemia and anorexia, it significantly negatively affects T-cell functions [47]. Elevated levels of IL-6 are often observed in advanced lung cancer patients, which, at diagnosis, frequently present cachexia syndrome, an inflammatory driven severe condition characterized by involuntary weight-loss accompanied by chronic inflammation, fatigue, anorexia, and anemia, where IL-6 is actually one of the key pathogenetic mediators [47]. Thus, although IL-6 initially participates in the activation of the immune response, its prolonged, chronic release ultimately contributes to immunosuppression, severe cancer-related symptoms, and poor general patient status and prognosis. Consistent with the above evidence, blocking chronic inflammation, primarily driven by IL-6, may be fundamental in improving the efficacy of currently available immunotherapy, especially in advanced lung cancer patients [25].

#### 2.1.2. Role of EGFR Mutations in Influencing TME, TAM Polarization, and Response to Immunotherapy

**Figure 2:**
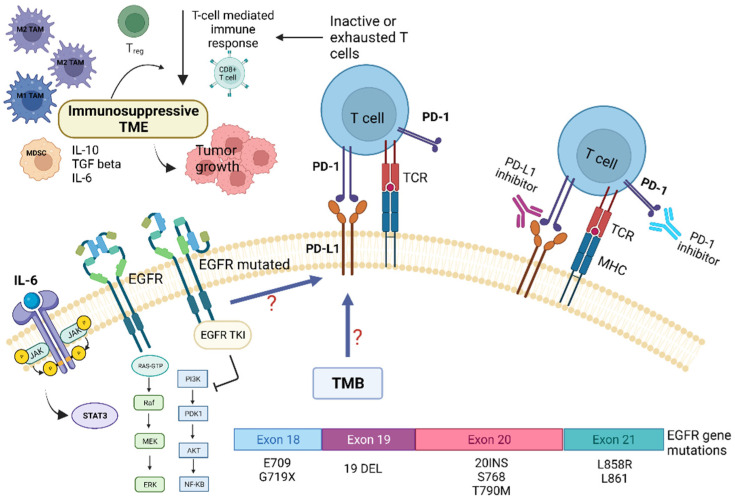
Role of tumor microenvironment in EGFR-mutated NSCLC in influencing resistance pathways to targeted TKI treatment and potential targets for immunotherapy. EGFR mutations are associated with immunosuppressive TME, lower tumor-mutation burden (TMB), and increased PD-L1 expression. EGFR mutations may promote cancer immune escape through modulation of the PD-1/PD-L1 pathway, which in turn determine T-cells inactivity and/or exhaustion. This also leads to EGFR-TKI resistance. In addition, EGFR mutations influence several TME components, such as tumor-infiltrating lymphocytes (TILs), Tregs, MDSCs, TAMs, and immunoregulatory/proinflammatory cytokines, i.e., IL-6. The latter, through the activation of the STAT-3 intracellular pathway, contribute to tumor growth and resistance to targeted therapies. Abbreviations: AKT—serine-threonine kinase; EGFR, epidermal growth factor receptor; ERK—extracellular signal-regulated kinase; IL—Interleukin; JAK—Janus kinase; MHC—major histocompatibility complex; MEK—mitogen-activated protein kinase; MDSC—myeloid-derived suppressor cells; NF-kB, nuclear factor kappa B; PI3K—phosphatidylinositol-4,5-bisphosphate 3-kinase; PD-1—programmed death; PD-L1—programmed death ligand-1; TKI—Tyrosine kinase inhibitors; Treg—regulatory T-cell; STAT3—signal transducer and activator of transcription 3; TCR—T-cell receptor; TMB—tumor mutational burden. Created with BioRender.com (https://biorender.com/, accessed on 17 May 2022).

Preclinical and clinical studies have pointed out that the TME of patients with NSCLC harboring EGFR mutations displays peculiar characteristics and may modulate the antitumor immune response [6]. Several trials indicated that EGFR mutations are associated with immunosuppressive TME, lower tumor mutation burden (TMB), and increased PD-L1 expression [2,6]. TMB is defined as the total number of substitution, insertion, and deletion mutations per megabase of the coding region that encodes a tumor gene. Recent studies suggested that reduced TMB may predict a poor response to immune checkpoint inhibitors (ICIs) in patients carrying EGFR mutations [6,7]. Preclinical studies indicated that EGFR mutations lead to cancer immune escape through the PD-1/PD-L1 pathway [6]. In addition, it has been shown that EGFR mutations influence TME components, such as tumor-infiltrating lymphocytes (TILs), Tregs, MDSCs, TAMs, and immunoregulatory cytokines (Figure 2).

A retrospective study observed that NSCLC tumors harboring EGFR mutations had low expression levels of PD-L1 and few CD8^+^ TILs. In contrast, other studies have detected high PD-L1 expression in this type of tumor [11]. Preclinical studies have demonstrated that EGFR activation upregulates intrinsic PD-L1 expression, inducing T-cell apoptosis and immune escape in EGFR-mutated NSCLC. In a genetically engineered mouse model of lung adenocarcinoma carrying an EGFR mutation, decreased macrophage MHC-II expression, enhanced macrophage IL1RA expression, and increased macrophage phagocytic activity have been observed and attributed to the M2 macrophage phenotype [48]. The presence of inflamed TME is considered a positive predictive factor for the response to immunotherapy. Although EGFR-mutated NSCLC typically is not associated with inflamed TME, characterized by low levels of CD8^+^ T cells and immune-suppressive cells, the numbers of Tregs and PD-L1 expression levels are increased in this cancer (Figure 2).

This leads to reduced effector T-cell activity and promotes TME favoring immune escape and cancer progression [48,49]. In EGFR-mutated cancers, TME displays high Treg infiltration without CD8^+^ T-cell infiltration. The recruitment of effector CD8^+^ T cells is prevented by the downregulation of CXCL10 through IRF1. In contrast, the stimulation of Treg infiltration is achieved through the upregulation of CCL22 through JNK/-JUN. Such immunological status may correlate with resistance to immunotherapy. Moreover, in EGFR-mutated cancers, the non-inflamed immunosuppressive TME (high levels of Tregs and low levels of CD8^+^ T cells) diminishes the expression of EGFR by Tregs, leading to the development of the resistance to TKIs. In conclusion, in the TME of EGFR-mutated NSCLC, high Treg infiltration occurs in the context of the non-inflamed TME. Therefore, EGFR mutations play a crucial role in cell growth, survival, and development of immune escape mechanisms [48].

#### 2.1.3. Dynamic Changes of the TME during TKI Treatment

**Figure 3:**
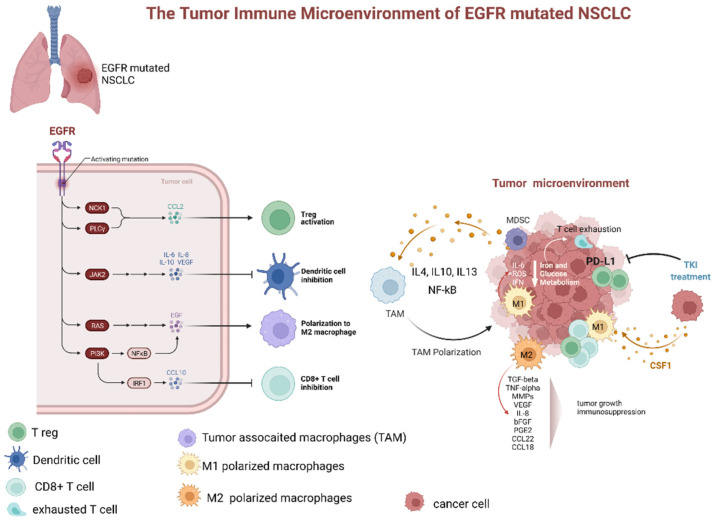
Dynamic changes of tumor microenvironment (TME) of EGFR-mutated NSCLC during tyrosine kinase inhibitor treatment. TME of EGFR mutated adenocarcinoma is typically characterized by prevalence of M2 polarized macrophage, low levels of CD8+ cells, increased number of Treg, and upregulation of PD-L1. The latter, especially if associated with macrophage-mediated inflammation particularly through IL-6 and increased ROS levels, contributes to T-cell exhaustion. Additionally, several factors secreted by M2 polarized TAMs (as TGFbeta, TNFalpha, MMPs, VEGF, IL-8, bFGF, PGE2, CCL22, and CCL18). These factors contribute to tumor progression and immunodepression. The TKI treatment has been associated with a decrease in PD-L1 expression, lowering of Treg, and promotion of TAM polarization from M2 to M1 phenotype. Abbreviations: EGFR—epidermal growth factor receptor; NSCLC—non-small cell lung cancer; JAK—Janus Kinase; PI3K—phosphatidylinositol-3 kinase; NF-kB—nuclear factor-κB; IRF1—interferon regulatory factor 1; IL—interleukin; TAM—tumor-associated macrophages; MDCS—myeloid-derived suppressor cells; PD-L1—programmed death-ligand 1; ROS—reactive oxygen species; IFN—interferon; TKI—tyrosine kinase inhibitor; CSF1—colony stimulating factor 1; TGF—tumor growth factor; TNF—tumor necrosis factor; MMP—matrix metalloproteases; VEGF—vascular endothelial growth factor; PGE—prostaglandin E; CCL—C-C-motif ligand. Created with BioRender.com (https://biorender.com/, accessed on 17 May 2022).

Treatment with EGFR-TKIs alters the TME and decreases PD-L1 expression levels, which may also affect the response to immunotherapy. Additionally, EGFR-TKIs regulate the strength of the immune response through TME changes (Figure 3). In particular, EGFR-TKIs increase the presentation of MHC class I and II molecules and potentiate T-cell-mediated tumor killing. Moreover, the numbers of tumor-infiltrating effector Tregs were significantly lower in patients treated with TKIs. The lung cancer TME contains CD8^+^ T cells and immune-suppressive TAMs expressing PD-L1. From a clinical standpoint, the presence of PD-L1^+^ TAMs may predict the effectiveness of ICIs.

Although according to one clinical trial, pembrolizumab did not elicit a significant response in patients with EGFR-mutated lung cancer naïve for EGFR-TKI, even in the presence of high PD-L1 expression, the efficacy of this ICI could be influenced by TME changes during the EGFR-TKI treatment [50,51]. Recently, IT effectiveness was shown to correlate positively with the number of CD8^+^ lymphocytes and negatively with the number of FOXP3^+^ tumor-infiltrating lymphocytes in patients who acquired resistance to EGFR-TKIs [52]. In another study, both mouse and human macrophages were demonstrated to prevent killing of cancer cells by CD8^+^ T-cells, thereby affecting the response to immunotherapy [53]. Nonetheless, according to lung cancer clinical data from case series and clinical trials, the presence of TAMs expressing PD-L1 apparently correlates with a good response to immunotherapy, owing to the negative effects on cytotoxic lymphocytes [19,54,55,56]. A retrospective study evaluated the effectiveness of immunotherapy in patients with EGFR-mutated NSCLC by assessing both PD-L1 expression and TME parameters, including numbers of CD8^+^ TILs [57]. On the basis of PD-L1 expression levels and abundance of CD8^+^ TILs, the TME was divided into four subtypes: type I—adaptive immune resistance (PD-L1^+^/CD8^+^); type II—immune ignorance (PD-L1^−^/CD8^−^); type III—intrinsic induction (PD-L1^+^/CD8^−^); and type IV—immune tolerance (PD-L1^−^/CD8^+^) [58]. The results of that study confirmed that TKIs alter PD-L1 and PD-L2 expression levels and affect the numbers of CD8^+^ TILs. High abundance of CD8^+^ TILs was shown to be associated with favorable outcomes in EGFR-mutated NSCLC. Furthermore, high levels of CD8^+^ TILs may affect the response to EGFR-TKI treatment [57]. Su et al. [59] reported a high proportion of PD-L1^+^/CD8^+^ cases among patients with de novo resistance to first-line EGFR-TKIs for advanced NSCLC. These findings suggested that NSCLCs with high PD-L1 expression and large numbers of CD8^+^ TILs benefit less from TKI treatment despite EGFR mutations. In addition, many reports indicated that relatively high PD-L1 expression in EGFR-mutated NSCLC was related to lower response to EGFR-TKIs and worse progression-free survival [57,60,61].

In recent clinical trials, it has been observed that ICIs promote macrophage polarization from the M2 to M1 phenotype [19]. In a retrospective study evaluating the relationship between TAMs and response to EGFR-TKIs in treatment-naïve patients, irrespective of the EGFR mutation status, both univariate and multivariate analyses showed that TAMs and EGFR mutations were independent prognostic factors of survival. However, as proposed in the review by Biswas et al. [62], these parameters correlate with each other. Tumors carrying EGFR mutations had higher TAM counts than tumors with wild-type EGFR (90% vs. 38.5%). Hence, TAM counts may predict the response to EGFR-TKIs, as TAMs contribute to drug resistance induced by the activity of stromal fibroblasts, as previously demonstrated by Wang et al. in vitro and in vivo [63]. According to the data obtained by two studies that evaluated patients with early and advanced NSCLC, host immunosurveillance is unimpaired in the early stages of NSCLC, when the M1 macrophage phenotype prevails, but falls apart in the advanced stages due to M2 phenotype polarization [14]. Targeted therapy blocks specific signaling pathways, whereas immunotherapy stimulates the immune system to attack tumor cells that previously evaded immune surveillance [64]. Subgroup analysis of clinical trials showed no survival benefit from immunotherapy in patients carrying EGFR mutations [65,66]. EGFR activation increases PD-L1 expression in tumor cells, inducing T-cell apoptosis and immune escape [64]. EGFR-TKIs strengthen MHC class I and II antigen presentation in response to IFN-γ, increasing T-cell-mediated tumor killing [67,68]. These findings explain the potential synergistic effects of immunotherapy and TKIs. However, the clinical benefits of such a combination may be limited [64]. Non-inflamed tumors lack significant lymphocyte infiltration, exhibit low PD-L1 expression, and have elevated levels of immunosuppressive elements in the TME, so such tumors may not be particularly sensitive to immunotherapy [69,70]. EGFR-TKIs modify the TME, weakening the suppressive activity of Tregs and promoting activity of cytotoxic T-cells [64]. EGFR-TKIs were demonstrated to boost the levels of cytotoxic CD8^+^ T cells and DCs, eliminate FOXP3^+^ Tregs, and inhibit macrophage polarization to the M2 phenotype, albeit only for a short time. However, EGFR-TKIs also decrease PD-L1 expression in cancer cells. Therefore, a combination of TKIs and immunotherapy may have suboptimal synergistic effects. Tariq et al. observed that inhibition of the STAT6 pathway by gefitinib prevented M2 polarization, but no dynamic changes during TKI treatment were evaluated [71]. Nevertheless, after a long period of TKI treatment, IL-10 activated the STAT3 pathway in MDSCs, inducing Treg activity, inhibiting DCs, and increased M2 macrophage polarization. Thus, the initial effect of gefitinib was neutralized [64].

#### 2.1.4. Rationale for Combining Immunotherapy and TKI Treatment to Overcame Resistance

Based on the above evidence, it can be hypothesized that a combined treatment with ICIs and EGFR-TKIs could increase anticancer activity. Some studies have consistently suggested a synergistic effect of a combination of PD-1/PD-L1 inhibitors with EGFR-TKIs in EGFR-mutated NSCLC with PD-L1 overexpression [72,73]. However, data regarding the combined use of PD-1/PD-L1 inhibitors and EGFR-TKIs are controversial [74]. Combined therapy using TKIs and ICIs is currently being explored, but the toxicity of these drugs may preclude their concomitant use [75,76,77,78]. However, in a small study, a combination of the TKI erlotinib and ICI nivolumab appeared to be safe and well tolerated [79]. Notably, erlotinib was shown to increase MHC I antigen presentation, rendering cancer cells vulnerable to T cells [80]. Furthermore, two studies showed that TKI downregulated PD-L1 expression and modulated T-cell-mediated immune responses [72,81,82]. EGFR TKIs also had immunostimulatory activities, as reported by Venugopalan et al. [83] and Jia et al. [64]. Moreover, in a study in mouse models of lung cancer with EGFR mutation, the TKI erlotinib was demonstrated to boost leukocyte infiltration and enhance antigen-presenting function [83]. Hence, TKI treatment is thought to re-establish a healthy immune microenvironment, inducing tumor regression. However, a combination of TKI therapy with immunotherapy does not prevent disease relapse [80].

Teng et al. [58] assessed the effectiveness of immunotherapy using a TME model based on TIL numbers and PD-L1 expression levels. Their findings suggest that tumors with the immunoinflammatory TME (PD-L1^+^ and TIL^+^) may benefit greatly from ICI treatment. Further, lower CD8^+^ TIL levels were related to EGFR mutation. EGFR mutations can upregulate CD73 expression, increase Treg numbers, and stimulate conversion of ATP into immunosuppressive adenosine, contributing to cancer progression and metastasis [8,84,85]. Increased activation of Tregs mediated by adenosine and accumulation of MDSCs, along with lower anticancer activities of natural killer cells and DCs, increased macrophage polarization toward the M2-phenotype, and inhibition of the effector T-cell-mediated response collectively led to tumor immune escape [86]. Preclinical and clinical studies demonstrated that the application of EGFR-TKIs increases MHC expression and induces FOXP3 degradation, leading to reduced Treg activity and infiltration in the TME [87]. Furthermore, EGFR-TKIs boost T-cell-mediated anticancer function, reduce T-cell apoptosis, inhibit M2 polarization, and enhance IL-10, CCL2, and INF-γ levels. CCL2 promotes differentiation of T cells into Th2 cells that have anti-inflammatory functions. This, in turn, activates the STAT3 pathway in MDSCs, which migrate to the TME and exert immunosuppressive activity. Furthermore, MDSCs promote angiogenesis by stimulating the secretion of the vascular endothelial growth factor, and release of MMPs [8].

In conclusion, the influence of EGFR-TKIs on the TME in EGFR-mutated adenocarcinoma may play a critical role in the response to immunotherapy.

#### 2.1.5. Overcoming Resistance and Potentiate Response to Immunotherapy in Patients with EGFR-Mutated NSCLC by Targeting TAM Related Inflammation and Cytokine Storm

TAMs have been highlighted as one of the major components of the immunosuppressive TME and, consequently, are an attractive target to improve responses to immunotherapy. Several strategies, such as TAM depletion, TAM reprogramming, and targeting of TAM functional molecules, have been proposed to enhance the efficacy of ICIs. Preclinical studies carried out in mouse models of several solid tumors, including lung cancer, suggest that a combination of these strategies with immunotherapy can enhance therapeutic responses, but all of these strategies need further investigation before they may be applied in clinical practice [88]. In lung cancer, ICIs targeting the PD-1/PD-L1 pathway have shown limited success in patients with NSCLC that harbored activating EGFR mutations, because activated EGFR signaling allows NSCLC cells to use multiple strategies to create an immunosuppressive TME, including recruitment of TAMs and Tregs, and at the same time, produce inhibitory cytokines and metabolites [89]. Therefore, some studies have explored novel mechanisms to reverse the suppressive TME and consequently improve the efficacy of ICIs in such patients. A recent study by Chen et al. [90] showed that activated EGFR-signaling induced ILT4 overexpression in NSCLC cells via the ERK1/2 and AKT signaling pathways and suppressed tumor immunity by recruiting M2-like TAMs, diminishing the T-cell response. EGFR activation observed in patients with NSCLC has two mechanisms: intrinsic activation caused by EGFR mutations and extrinsic activation by ligand recruitment in patients with wild-type EGFR [91]. The observations by Chen et al. [90] suggest that ILT4 inhibition prevents immunosuppression and tumor growth. In fact, they demonstrated that ILT4 inhibition reversed the immunosuppressive features of the TME, so this approach might be a promising strategy for the second-line treatment in both TKI-resistant EGFR-mutated and EGFR wild-type NSCLC. Furthermore, ILT4 inhibition may also help to overcome resistance to ICIs in this class of patients [90]. Recently, Tu et al., showed that in a xenograft mouse model of EGFR-mutated NSCLC, neither anti–PD-L1 nor anti-CD73 antibody alone inhibited tumor growth compared with the effect of the isotype control. However, a combination of both antibodies significantly inhibited tumor growth, increased the number of tumor-infiltrating CD8^+^ T cells, and enhanced IFN-γ and TNF-α production by these T cells. Consistently, the authors observed an increase in expression levels of genes involved in inflammation and T-cell function in tumors treated with a combination of anti-PD-L1 and anti-CD73 antibodies. These results indicate that a combination of anti-CD73 and anti-PD-L1 therapies may be effective in treating EGFR-mutated NSCLC [84].

## 3. Discussion

ICIs that block the PD-1/PD-L1 pathway have revolutionized the clinical care of patients with locally advanced or metastatic NSCLC [92]. However, patients with EGFR-mutated NSCLC benefit less from the anti-PD-1/PD-L1 treatment than patients without the mutation [7]. However, the mechanism underlying the resistance to anti-PD-1/PD-L1 agents in EGFR-mutated NSCLC remains unclear. Some authors have demonstrated lower IFN-γ levels and decreased T-cell infiltration in EGFR-mutated NSCLC [69], which suggests decreased immunogenicity or suppression of the immune response in the TME. Given that PD-L1 expression in tumor tissue is an important biomarker that predicts clinical outcomes of the anti-PD-1/PD-L1 treatment [93], it is possible that tumors in EGFR-mutated NSCLC express low levels of PD-L1. A pooled analysis of 15 published studies and data from the Cancer Genome Atlas showed that patients with NSCLC harboring EGFR mutations have lower PD-L1 expression in their tumor tissue [69]. At the same time, other studies have demonstrated upregulation of PD-L1 in NSCLC with activating EGFR mutations [94,95]. Because reports on PD-L1 status in EGFR-mutated NSCLCs are conflicting [96], there may be other mechanisms at play that contribute to immunosuppression in the tumors of such patients. The TME plays a key role in regulating tumor progression and significantly affects the efficiency of immune response in patients with mutated EGFR. As we discussed in this review, evidence from the literature supports the notion that the TME in EGFR-mutated NSLC is immunosuppressive, with reduced TMB, low expression of PD-L1, low TIL numbers, and high Treg infiltration. Additionally, NSCLC tumors harboring EGFR mutations typically present with non-inflamed TME, which has been associated with a poor response to immunotherapy. In this regard, it remains unclear whether there are differences in the TME and ICI efficacy in NSCLC with different EGFR mutation subtypes. Two recent studies have indicated that from an immunological perspective, oncogenic mutations may be an important factor for cellular immune-suppression [97,98]. Several potential approaches to improve the response to immunotherapy have been tested. Targeting TAM and DC therapy may be an interesting future direction for patients with EGFR mutations. In addition, combining ICI with TKI may be another effective therapeutic strategy. In any case, when considering the mechanisms that modulate the effectiveness of immunotherapy, in addition to establishing the oncogenic mutations, the evaluation of the patient’s general status is crucial. This is especially important in lung cancer patients who, at diagnosis, often present with a compromised general status, with cachexia, chronic inflammation, and consequent immunodepression. Indeed, these factors may be particularly important in explaining the inferior response to immunotherapy in some subsets of patients with advanced-stage cancer [25]. Immunotherapy may be combined with drugs that modulate chronic inflammation, counteract oxidative stress, and correct disturbances of energy and iron metabolism, which significantly impact the efficiency of the immune system. In this regard, several years ago, we tested the efficacy of a combination treatment with immunotherapy (recombinant IL-2), anti-inflammatory agent (medroxyprogesterone acetate), and antioxidants in patients with advanced cancer [99,100].

## 4. Conclusions

In conclusion, different immunosuppressive mechanisms, including—but not limited to—altered TME, are involved in the resistance to immunotherapy in EGFR-mutated NSCLC. These mechanisms should be properly characterized and targeted by a combined pharmacological approach. In this context, the specific processes that regulate the effectiveness of the immune response in relation to the disease stage, cancer-related inflammation with related systemic symptoms, and patient general status should not be underestimated.

## Data Availability

Not applicable.
